# Gold@MnFe‐Prussian Blue Analog Yolk@Shell Nanoparticles for Light‐Triggered and pH‐Sensitive Drug Release

**DOI:** 10.1002/smll.202514869

**Published:** 2026-02-19

**Authors:** Shoaib Azeem, Javier Alda‐Gómez, Roger Sanchis‐Gual, Marc Coronado‐Puchau, Eugenio Coronado Miralles

**Affiliations:** ^1^ Instituto de Ciencia Molecular Universitat De València Paterna Spain

**Keywords:** drug delivery, gold, nanoparticles, Prussian Blue Analogs, yolk@shell

## Abstract

Yolk@shell nanostructures have emerged as promising platforms for biomedical applications due to their unique architecture, which combines the high surface area and loading capacity of hollow nanoparticles with the structural stability and functional versatility of core@shell systems. In this work, we report the synthesis of multifunctional Au@MnFe‐Prussian Blue Analog (PBA) yolk@shell nanostructures through a two‐step process: initial formation of Au@PBA core@shells, followed by selective leaching of the inner PBA shell. This process not only generates a well‐defined internal cavity of ∼75 nm, enhancing drug‐loading capacity, but also induces partial etching of the gold core and its redeposition onto the PBA shell. This redistribution of gold leads to the emergence of absorbance in the near‐infrared (NIR) region, enabling efficient photothermal conversion. Meanwhile, the MnFe PBA shell offers pH‐sensitive properties and excellent biocompatibility. Using doxorubicin (DOX) as a model chemotherapeutic, the system exhibits a loading efficiency of ∼50% and a pH‐dependent release profile, with enhanced release (∼150%) under NIR irradiation. *In vitro* studies demonstrate effective cellular uptake and synergistic cytotoxicity upon combined chemo‐ and photothermal treatment. This study reveals the significant potential of Au@MnFe PBA yolk@shell architectures for controlled drug delivery.

## 1 Introduction

Yolk@shell nanostructures have attracted considerable attention as multifunctional nanoplatforms due to their unique architecture, which features a core encapsulated within a hollow shell. This structural design, intermediate between hollow and core@shell nanoparticles (NPs), combines the advantages of both structures together, offering enhanced stability, increased surface area, and improved loading and release capabilities [1, 2, 3, 4]. Notably, by combining complementary materials, these heterostructures can integrate a wide range of properties and offer improved performance over conventional core@shell or hollow NPs [5, 6]. Indeed, this versatility and multifunctionality make them particularly advantageous for drug delivery applications, where precise control over loading capacity, release kinetics, and targeting efficiency is crucial for therapeutic success [7, 8].

In this context, Au NPs and Prussian Blue Analogs (PBAs) stand out as excellent building blocks for yolk@shell designs due to their complementary properties [9, 10, 11, 12]. Au NPs exhibit strong light absorption through localized surface plasmon resonance (LSPR), which enables efficient photothermal therapy by converting light into heat for localized tumor ablation [13]. In addition, they can also be functionalized with many targeting ligands. However, their drug‐loading capacity is inherently very limited. In contrast, PBA‐coordination polymers with the general formula A_a_M^II^_x_[M’^III^(CN)_6_]ᵧ·nH_2_O offer high drug‐loading potential, pH‐responsive release behavior, effective intrinsic imaging capabilities as a contrast agent while exhibiting excellent biocompatibility [14, 15, 16]. In particular, MnFe‐based PBAs have demonstrated notable potential in drug delivery due to their hollow morphology and their ability to release chemotherapeutic drugs such as doxorubicin (DOX) in acidic tumor environments [17, 18, 19].

However, the synthesis of well‐defined Au@PBA structures ‐particularly with yolk@shell architectures‐ remains challenging due to the susceptibility of Au to cyanide‐induced etching during PBA formation [20]. To date, only one yolk@shell Au@PBA nanostructure with a narrow cavity has been reported, using Au@Ag nanocubes as templates. In that approach, the Ag shell was partially etched and replaced with PBA precursors [21]. Nevertheless, this approach faces limitations, including incomplete Ag removal and the exclusive formation of PB shells. These challenges underscore the need for alternative synthetic strategies to achieve structurally robust, multifunctional Au@PBA yolk@shell NPs.

**SCHEME 1:**
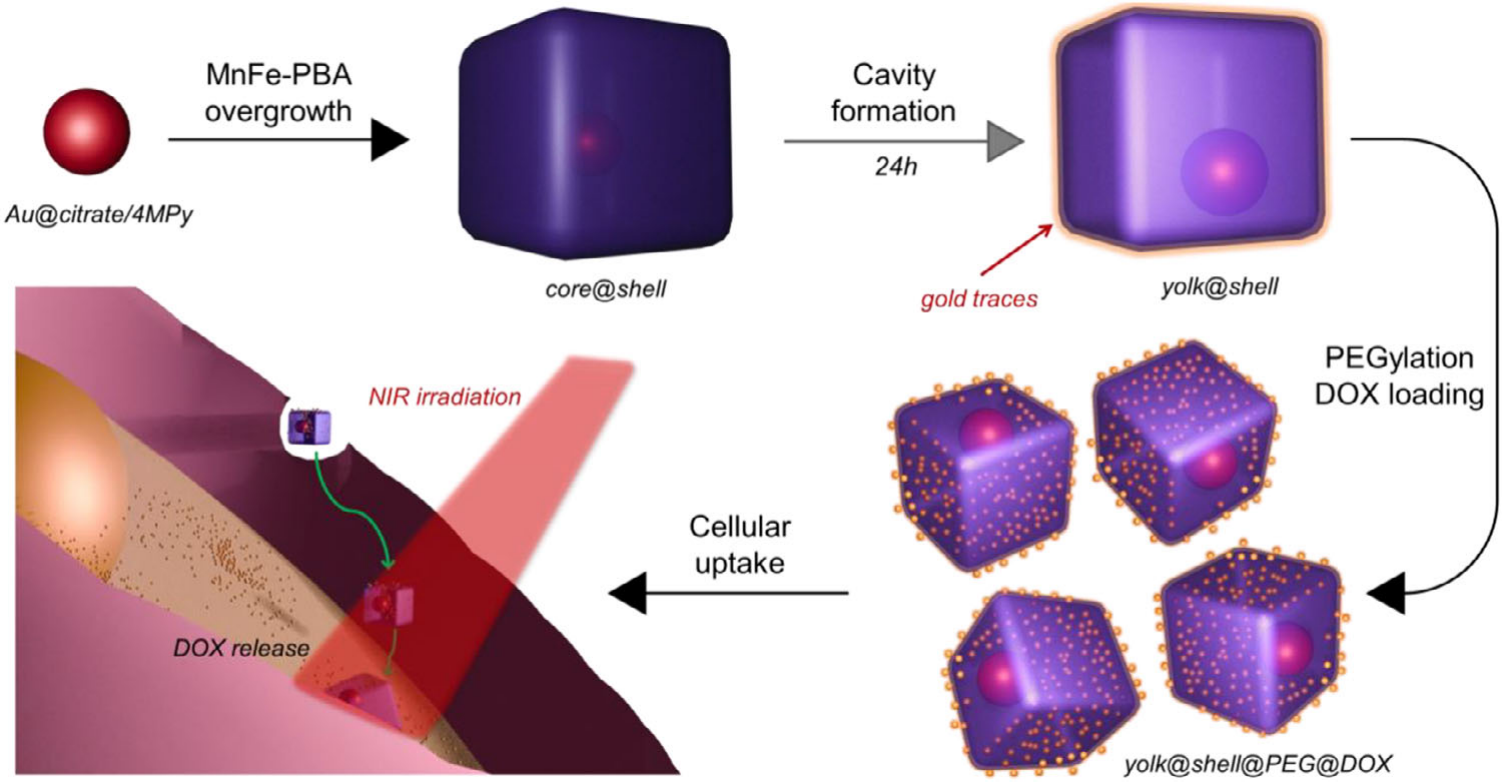
Illustration of the synthesis of PEGylated yolk@shell Au@MnFe‐PBA NPs, their DOX loading and intracellular drug release. After cellular internalization, photothermal heating induced by NIR irradiation accelerates DOX release for enhanced therapeutic efficacy.

In this work, we report a novel strategy for preparing Au@MnFe yolk@shell nanostructures composed of a gold NP core encapsulated within a hollow MnFe PBA shell. Structural and spectroscopic analyses confirm the formation of a well‐defined internal cavity of around 75 nm, attributed to selective leaching of the inner PBA during the synthesis. This yolk@shell design effectively integrates in a single nanostructure the optical properties of gold with the pH‐responsive and drug‐loading capabilities of MnFe PBAs, offering a versatile platform for biomedical applications (Scheme 1). DOX was employed as a model chemotherapeutic to assess the drug delivery performance of the system. The Au@MnFe yolk@shell NPs exhibit high drug loading efficiency of 50% and a pH‐sensitive release profile, achieving *ca*. 30% at pH 5 after 6 h. Moreover, near‐infrared (NIR) laser irradiation (808 nm, 10 min) significantly enhanced DOX release (∼150% increase in the first hour), attributed to photothermal features of Au. These findings highlight the potential of Au@MnFe yolk@shell NPs as multifunctional platforms for NIR‐ and pH‐triggered drug delivery in cancer therapy.

## 2 Results and Discussion

To synthesize Au@MnFe yolk@shell NPs, we adapted a protocol previously reported by our group [22] in which 4‐mercaptopyridine (4‐MPy) molecules are anchored onto Au NPs. This surface functionalization facilitates the controlled growth of a MnFe PBA shell. In this manner, the CsMnFe PBA was formed by the simultaneous and slow addition of the precursors (MnCl_2_·4H_2_O, CsCl, and K_3_Fe(CN)_6_) into the colloidal solution of 4‐MPy‐modified Au NPs, followed by continuous stirring for 24 h. Cs ions were incorporated as counter cations, as their inclusion within the MnFe PBA lattice has been reported to enhance structural stability [23] and improve imaging contrast in biomedical applications [24]. Notably, in the absence of Cs, the PBA structure tends to dissolve rapidly, potentially leading to a premature and uncontrolled drug release.

**FIGURE 1:**
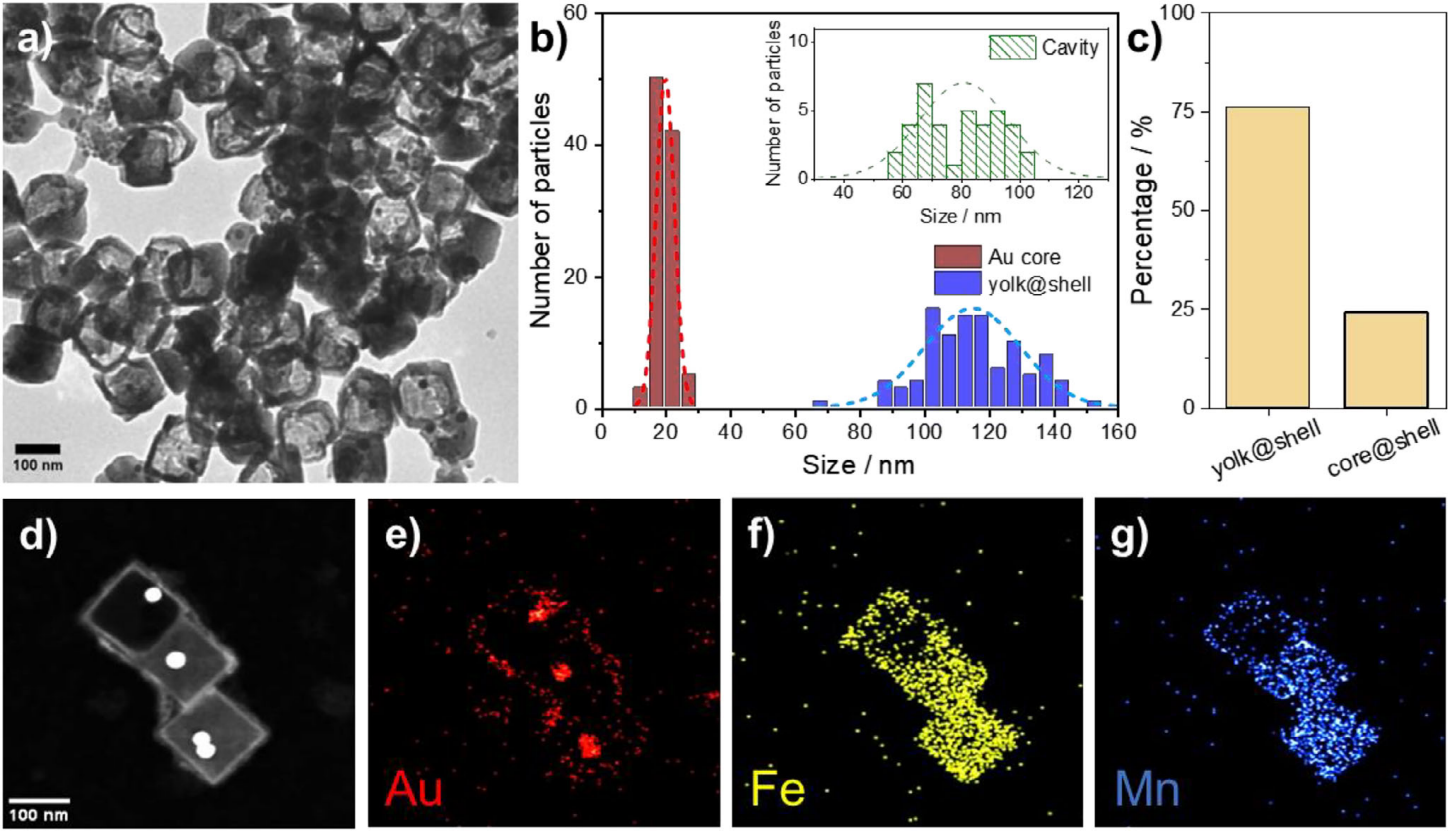
Yolk@shell NP characterization. a) TEM image of the synthesized yolk@shell NPs. b) size distribution histogram showing the size of the Au core, the internal cavity, and the overall NP diameter. c) Percentage of NPs exhibiting the yolk@shell morphology. d‐g) EDX mapping for the Au, Mn, and Fe.

Transmission electron microscopy (TEM) images confirmed the successful formation of yolk@shell nanostructures. As shown in Figure 1a, the obtained NPs consist of a single Au core enclosed within a well‐defined hollow cavity surrounded by a PBA shell. Size analysis (Figure 1b) revealed a narrow distribution, with an average core diameter of *ca*. 20 nm, an internal cavity of *ca*. 75 nm, and an overall NP diameter of *ca*. 115 nm. Around 75% of the NPs exhibited the desired yolk@shell architecture (Figure 1c), while the remaining particles presented either core@shell morphologies or partially damaged or incomplete shells. Interestingly, traces of Au were also detected within the shell of the yolk@shell NPs by energy dispersive X‐ray spectroscopy (EDX) mapping (Figure 1d–g), pointing towards a partial etching and redeposition of Au during shell growth. Additional EDX mapping and line‐profile analyses indicate that the amount of Au present on the PBA shell is relatively low compared to the core (Figure S1), suggesting that neither a continuous Au shell nor discrete Au NPs are formed on the surface.

To further investigate the elemental composition, inductively coupled plasma optical emission spectrometry (ICP‐OES) was performed on aggregates of the NPs. The overall chemical formula was determined to be approximately Au_0.37_@Cs_1.58_Mn_1_[Fe(CN)_6_]_0.84_. From this stoichiometry, the average oxidation state of Fe was calculated to be +2, based on charge balance among the Cs^+^ and Mn^2+^ cations and the [Fe(CN)_6_]^4−^ / [Fe(CN)_6_]^3−^ anions. This suggests that the [Fe(CN)_6_]^3−^ ions were reduced during synthesis.

**FIGURE 2:**
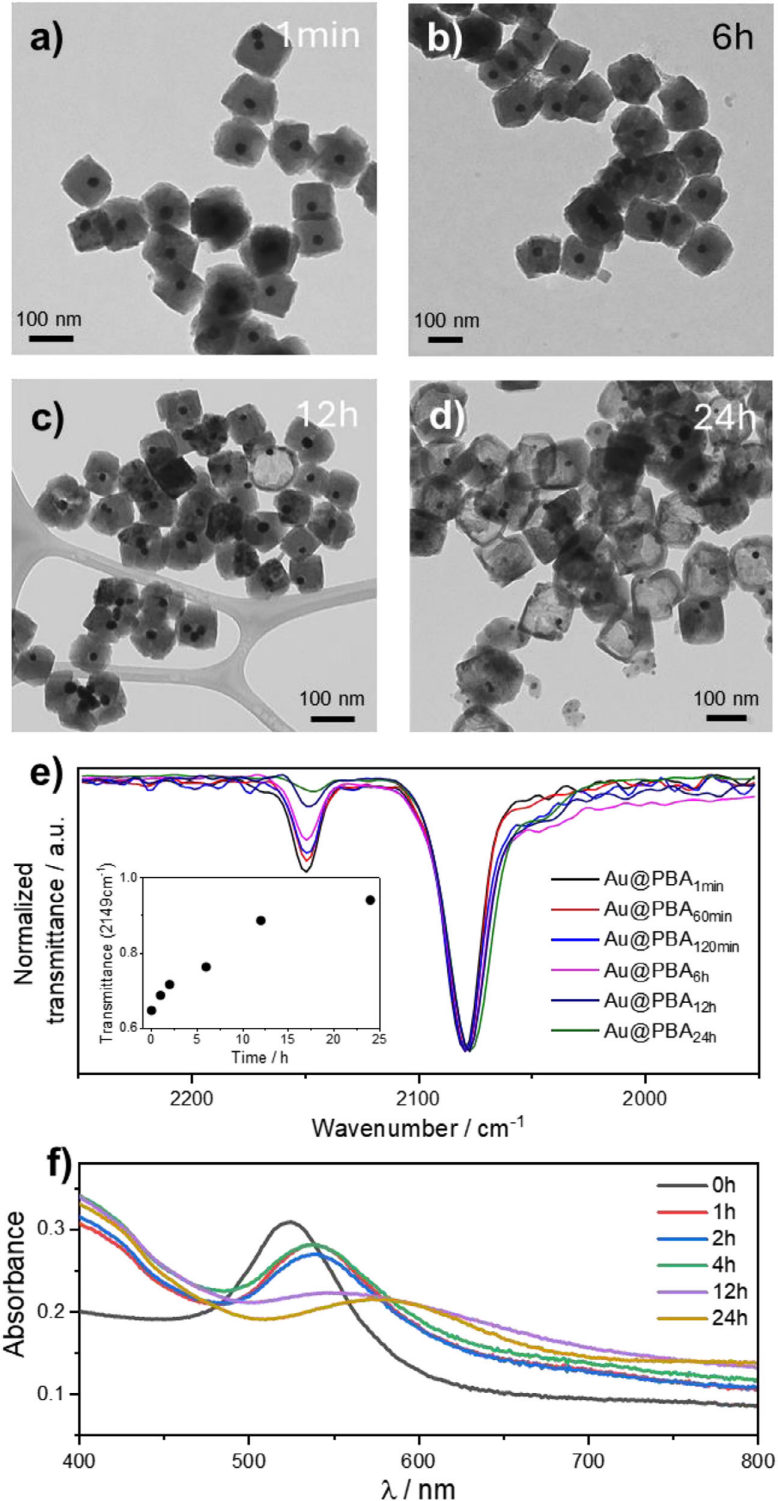
Time‐resolved formation of yolk@shell NPs. TEM images at different reaction times: (a) 1 min, (b) 6 h, (c) 12 h, and (d) 24 h. (e) ATR‐FTIR spectra of yolk@shell NPs recorded at different times, highlighting changes in the Mn–CN–Fe coordination environment during the hollowing process. f) UV‐Vis absorbance spectra of Au@MnFe nanoparticles at different reaction times.

To investigate the yolk@shell formation mechanism, we conducted a time‐resolved TEM study by collecting aliquots at various time intervals over a 24‐hour reaction period. TEM image in Figure 2a reveals the formation of uniform core@shells NPs within the first minute of the reaction. After 6 h, the morphology remains constant (Figure 2b) with only a slight increase in the particle size. Up to this point, no yolk@shell structure is observed. However, by 12 h the formation of small cavities within the particles begins to appear (Figure 2c). After 24 h, well‐defined yolk@shell heterostructures are clearly visible (Figure 2d), confirming a gradual transformation over time.

As has been reported [17, 25], citrate plays a crucial role in the hollowing process. In a series of syntheses with varying citrate concentrations, we observed that higher citrate levels promote the formation of more pronounced cavities, whereas lower concentrations result in more intact core@shell or solid NPs (Figures S2). These results demonstrate that citrate effectively modulates the extent of hollowing.

To gain further insight into the coordination environment of Mn–CN–Fe in the nanostructures, attenuated total reflectance Fourier‐transform infrared spectroscopy (ATR‐FTIR) was performed. As shown in Figure 2e, the characteristic peak at 2149 cm^−1^ corresponds to Mn(II)–Fe(III) pairs connected by CN^−^ ligands, while the band around 2080 cm^−1^ is attributed to Mn(II)–Fe(II) species. Over time, the intensity of the 2149 cm^−1^ band decreases relative to that at 2080 cm^−1^, indicating a preferential dissolution of the Mn(II)–Fe(III) coordination environment during the hollowing process. This behavior likely arises from the higher thermodynamic stability of Mn(II)–Fe(II) coordination, which results in its preferential preservation. As previously reported for hollow MnFe PBA compounds [25], the leaching of Mn(II) from Mn(II)–Fe(III) sites compromises the structural integrity of the shell, facilitating the progressive formation of an internal cavity.

Additionally, X‐ray photoelectron spectroscopy (XPS) was employed (Figure S3) to investigate the surface composition of the nanostructures. This analysis reveals that Mn(II)–Fe(II) species dominate the surface in both the initial core@shell and the final yolk@shell NPs. A small fraction of Fe(III) is detected in the core@shell structures, which is entirely absent in the yolk@shells. NPs collected after 12 h of reaction, corresponding to the stage when small cavities begin to form, exhibit an intermediate Fe(III) signal, decreased compared to the initial core@shell structures but not yet fully eliminated, while the Mn(II) signal remains unchanged. This suggests that Mn(II)–Fe(III) species reside primarily in the inner shell regions and are selectively leached during hollowing, whereas Mn(II)–Fe(II) species are preferentially preserved at the surface, contributing to the structural stabilization of the forming yolk@shell NPs.

To analyze the etching effect of free Fe(CN)_6_^3−^ on Au NPs [26], control experiments were performed in which Au NPs were incubated in 0.25 mM [Fe(CN)_6_]^3^^−^. Note that these conditions are similar to those used for the formation of yolk@shells, but no PBA is formed in this case, resulting in a higher concentration of free cyanide. UV–Vis measurements show only a partial decrease of the 520 nm plasmonic band after 24 h (Figure S4), indicating slow Au etching under these conditions. During yolk@shell formation, time‐resolved UV–Vis measurements reveal a progressive decrease of the Au plasmon band at *ca*. 520 nm, which is attributed to the etching of the Au core, accompanied by the emergence of a broad absorbance feature at 800 nm (Figure 2), suggesting that these two processes are correlated.

Based on these observations, we hypothesize that a portion of the gold core is etched by free Fe(CN)_6_^3−^/Fe(CN)_6_^4−^ and subsequently redeposited onto the shell. To investigate this redeposition process, control experiments were performed in which MnFe‐PBA NPs were synthesized in the presence of citrate and Au(CN)_2_^−^ ions. The resulting NPs exhibited hollow morphologies, and EDX mapping revealed the presence of Au atoms at the surface of the PBA shell (Figure S5), directly evidencing Au redeposition. This redeposition could be facilitated by redox processes as the oxidation of Fe(II) to Fe(III), which could promote the reduction of Au(I) to Au(0), enabling Au deposition at the shell surface.

XPS analysis of the Au4f region of the yolk@shells indicates that Au is present at the shell surface as a mixture of metallic Au and oxidized Au(I) species (Figure S6). Interpretation is limited by the low Au signal and overlap with Mn3s peaks, but the observed spectral broadening and peak positions clearly show that Au is not exclusively metallic. Considering the cyanide‐rich environment of the PBA shell, the XPS data are consistent with Au being present at the surface as a mixture of metallic Au and oxidized Au(I) species coordinated by CN^−^ ligands.

To probe the origin of the absorption at 800 nm, we carried out experiments mixing Au(CN)_2_^−^ with MnCl_2_ or Fe(CN)_6_^3−^. No new NIR absorption appeared after 24 h, indicating that soluble Au complexes are unlikely to account for the feature (Figure S7a). The reduction of MnFe(III) to MnFe(II) during hollowing, which decreases absorbance around 800 nm, also excludes this contribution (Figure S7b,c). Scattering effects were considered negligible, as scattering redistributes light rather than producing the observed broad absorption that is converted into heat (see below). Therefore, the 800 nm absorbance could arise from (i) aggregated Au clusters on the PBA shell, (ii) partially etched Au cores interacting with the shell, or (iii) the formation of multiple small Au cores during hollowing and etching processes.

The structure of the yolk@shell was analyzed and compared by power X‐ray diffraction (PXRD) with the core@shell and the pristine PBA (see experimental section for further details). As shown in the diffraction patterns in Figure S8, the CsMnFe PBA exhibits distinct peaks characteristic of its face‐centered cubic (fcc) structure. The successful formation of the MnFe PBA coating around the Au NPs is evident in both heterostructures. The core@shell structures display sharper and more intense peaks, indicating higher crystallinity. In contrast, the yolk@shell structures exhibit slightly broader peaks, possibly due to a smaller crystallite size resulting from the leaching process. A slight shift in peak positions in the core@shells (inset, Figure S8) suggests lattice strain or structural distortion near the Au/PBA interface [22]. In the yolk@shell, part of the strained PBA may have been removed during the leaching process, resulting in a reduction in the overall lattice distortion.

Following the formation of the yolk@shell NPs, surface functionalization was carried out to improve the colloidal stability and biocompatibility of the NPs [27]. This post‐synthetic modification was achieved by redispersing them in an aqueous solution of HS‐PEG‐COOH, as PEG can coordinate to the NP surface through its thiol and carboxylic acid groups [28, 29]. The successful PEGylation was confirmed by ATR‐FTIR (Figure S9), with characteristic PEG peaks observed in the fingerprint region (400–1600 cm^−^^1^) [30]. Thermogravimetric analysis (TGA) was carried out to quantify the PEG content. As shown in Figure S10, the PEGylated yolk@shell sample exhibits a mass loss near 300°C attributed to polymer decomposition, which is absent in the non‐PEGylated sample. As a result, a comparison of the residual masses of PEGylated and non‐PEGylated samples after heating up to ∼500°C indicates a PEG contribution to the final mass of ∼10%. Dynamic light scattering (DLS) was used to assess the colloidal stability of the PEG‐functionalized yolk@shell NPs in PBS. As shown in Figure S11, the hydrodynamic size and size distribution remain stable over a period of 48 h.

After functionalizing the NPs, drug loading was performed by dispersing the yolk@shell hybrids in a DOX solution. The presence of DOX was confirmed qualitatively via ATR‐FTIR and UV‐Vis spectroscopy (Figures S9 and S12). To quantify drug loading, the supernatant from the centrifuged DOX‐loaded NPs was analyzed. As shown in Figure S13a, an absorbance peak at 480 nm, characteristic of free DOX, was detected. Using a calibration curve (Figure S13b,c), the concentration of unincorporated DOX was determined. Thus, a higher absorbance correlates with lower loading efficiency. Based on this, the drug loading capacity was calculated as the mass of DOX incorporated per total nanocomposite mass, yielding a value of ∼50%. To validate this result, DOX‐loaded yolk@shell NPs were dissolved in DMSO and sonicated at 37°C for 50 min [31]. After centrifugation, the supernatant was analyzed (Figure S14a), and the released DOX content was quantified using a calibration curve (Figure S14b,c), confirming a loading efficiency of ∼47%, consistent with the previous method. It is important to note that drug loading was also performed on functionalized core@shell NPs, achieving a maximum value of 19% DOX (Figure S15), underscoring the role of the hollow cavity in increasing the surface area and enhancing drug loading capacity.

**FIGURE 3:**
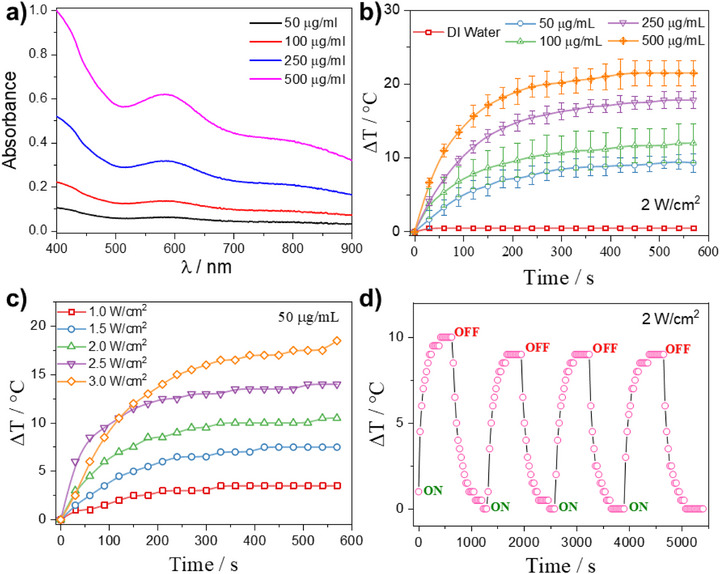
Photothermal performance of yolk@shell NPs. a) UV‐Vis absorbance spectra at various yolk@shell NP concentrations. b) Photothermal heating curves of yolk@shell NPs under 808 nm NIR laser irradiation (2 W/cm^2^) at different concentrations. c) Photothermal heating profile for 50 µg/mL yolk@shell NP irradiated at varying laser powers. d) Photothermal stability assessed by cyclic heating and cooling of 50 µg/mL NPs under repeated on/off NIR laser irradiation (2 W/cm^2^).

Furthermore, UV‐Vis‐NIR spectra were collected for yolk@shell NPs at concentrations of 50, 100, 250, and 500 µg/mL to explore their potential photothermal conversion. These absorbances were compared with core@shell and pristine MnFe PBAs. As shown in Figure 3a, the yolk@shell NPs exhibit an absorbance band around 800 nm, but no significant NIR absorbance was detected for either core@shell or the pristine PBAs (Figure S16). As localized heating can facilitate DOX release through hyperthermia [32, 33], yolk@shell NPs were irradiated with an 808 nm NIR laser (2 W/cm^2^, 10 min). In fact, a significant heating was observed, reaching a ΔT of ∼20°C at 500 µg/mL (Figure 3b), while for core@shell and pristine PBA samples this heating was minimal (<8°C, Figure S17).

Next, the effect of laser power on photothermal heating was evaluated at a fixed NP concentration of 50 µg/mL. A clear power‐dependent increase in temperature was observed (Figure 3c), with the highest power yielding a ΔT of ∼18°C. Notably, yolk@shell NPs demonstrated thermal stability and reproducibility over multiple on/off laser cycles (Figure 3d).

**FIGURE 4:**
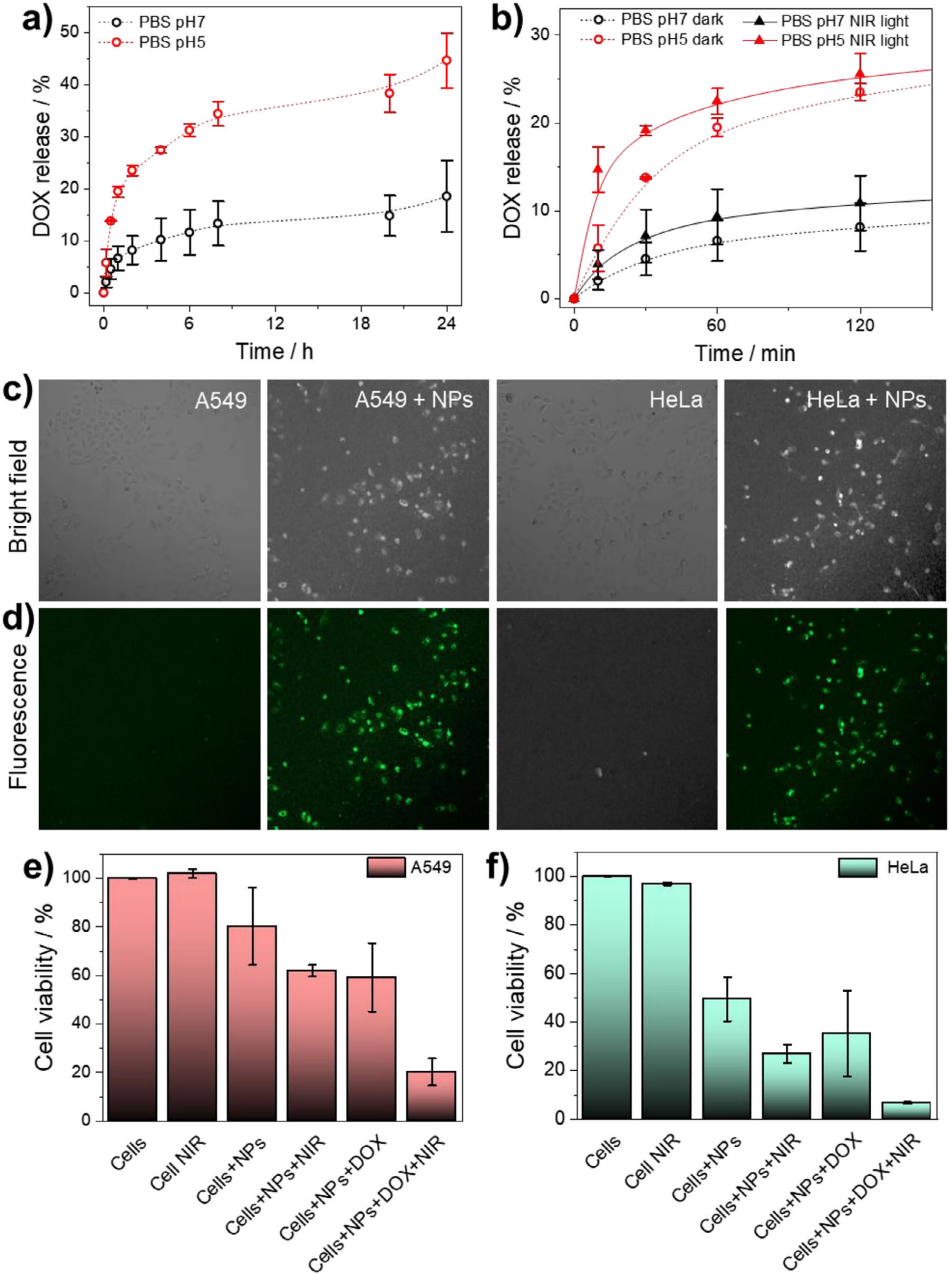
Dox release and cell viability: a) Cumulative DOX release from yolk@shell NPs in PBS at pH 7 and pH 5 over 24 h, b) DOX release profile during the first 2 h, comparing samples with and without 10 min of NIR irradiation (808 nm, 2 W/cm^2^), c,d) Confocal microscopy images (10x magnification) showing intracellular uptake of DOX‐loaded yolk@shell NPs (50 µg/mL) in A549 and HeLa cells after 4 h incubation, e,f) Cell viability of A549 and HeLa cells treated with 50 µg/mL yolk@shell NPs, with or without DOX, under dark conditions and after 5 min NIR laser exposure (2 W/cm^2^). Data are expressed as mean + SD of at least 3 technical replicates.

Subsequently, to examine DOX release of our novel nanocarrier, 1 mg of yolk@shell NPs was redispersed in 1 mL PBS at pH 7 and pH 5 (37°C), simulating physiological and tumor microenvironment conditions, respectively. Absorbance was measured at different time intervals over 24 h (Figure 4a). At pH 5, DOX release reaches ∼45%, compared to 15% values at pH 7, likely due to increased MnFe shell destabilization [34] and higher DOX solubility in acidic environments [35].

To assess the impact of photothermal stimulation, parallel samples were irradiated with an 808 nm laser (2 W/cm^2^, 10 min). At pH 5, a rapid release of *ca*. 15% is observed within the first 15 min (Figure 4b), representing a 150% increase with respect to non‐irradiated samples. This sharp enhancement is attributed to laser‐induced localized heating, which accelerates diffusion. However, by 24 h, DOX release in irradiated and non‐irradiated samples converged (Figure S18), indicating that NIR light primarily affects early‐stage release.

Encouraged by these results, we assessed cellular uptake in A549 and HeLa cells incubated with 50 µg/mL DOX‐loaded NPs for 4 h. Fluorescence microscopy confirms intracellular DOX localization, as evidenced by the green signal from DOX molecules observed within both A549 and HeLa cells (Figure 4c,d). TEM analysis (Figure S19) reveals that unloaded NPs accumulate in the cytoplasm, particularly within vesicles such as endosomes or lysosomes, consistent with cellular uptake via endocytosis.

Cytotoxicity assays were performed on A549 and HeLa cells treated with varying concentrations of yolk@shell NPs for 24 h. Unloaded NPs show very limited cytotoxicity toward A549 cells (viability >80% at 100 µg/mL), whereas HeLa cells are more sensitive (viability <65% above 50 µg/mL) (Figure S20). On the other hand, DOX‐loaded NPs cause significant viability reduction in both lines, with differences of ∼30% (A549) and ∼20% (HeLa) at 50 µg/mL compared to unloaded NPs. Subsequently, cells were treated with unloaded or DOX‐loaded yolk@shell NPs (preincubated for 4 h), followed by NIR irradiation (optimal irradiation conditions were set at 808 nm, 2 W/cm^2^, 5 min). As shown in Figure 4e,f, control cells exhibit minimal damage, confirming that laser irradiation alone is non‐toxic. However, cells treated with unloaded yolk@shell NPs show increased cell death due to the photothermal effect of Au. To sum up, cells exposed to DOX‐loaded yolk@shell NPs and NIR irradiation showed the greatest viability loss, highlighting a synergistic interaction between heat‐induced cytotoxicity and DOX chemotherapy. This synergy arises from enhanced intracellular DOX release triggered by photothermal heating, in combination with direct thermal damage, leading to improved therapeutic efficacy. Thus, these results demonstrate that yolk@shell Au@MnFe NPs are promising multifunctional nanostructures capable of acting as efficient chemotherapeutic carriers and photothermal agents upon NIR activation.

## 3 Conclusion

In this work, we have reported a novel synthetic approach for the preparation of yolk@shell nanostructures composed of a gold core and a hollow MnFe PBA shell. The transformation from core@shell to yolk@shell architecture is driven by the selective leaching of Mn(II) and Fe(III) ions, leading to the formation of a hollow cavity. Concurrently, partial etching and redeposition of gold onto the PBA shell led to the appearance of absorbance in the NIR region. This structural evolution results in a multifunctional nanoplatform that combines the NIR‐responsive photothermal properties of gold with the pH‐sensitive and high drug‐loading capacity of the hollow MnFe PBA shell.

Using DOX as a model chemotherapeutic agent, the yolk@shell NPs achieved a drug loading efficiency of 50%, significantly outperforming their core@shell counterparts (19%). Furthermore, in vitro studies demonstrated efficient cellular uptake and enhanced cytotoxicity resulting from DOX release. Notably, a synergistic therapeutic effect was observed when photothermal therapy was combined with chemotherapy, yielding greater cell death than either modality alone. Therefore, these results establish yolk@shell Au@MnFe NPs as a promising platform for stimuli‐responsive drug delivery and combinatorial cancer therapy, offering a versatile and effective strategy for future biomedical applications.

## 4 Experimental Section

### 4.1 Materials

All chemical reagents were purchased and used without further purification. Chloroauric acid, sodium citrate tribasic dihydrate, 4‐mercapto pyridine 95%, cesium chloride, manganese (II) chloride tetrahydrate, potassium hexacyanoferrate, Doxorubicin hydrochloride (pharmaceutical secondary standard), phosphate buffered saline (PBS), sodium hydroxide and HS‐PEG3500‐COOH were purchased from Sigma–Aldrich. Ultrapure water (18.2 MΩ) was used in the following syntheses.

### 4.2 Synthesis of NPs

#### 4.2.1 Au NPs

The synthesis was performed following the standard Turkevich's method [36]. To a 1 L boiling solution of 0.25 mM HAuCl_4_, 735 mg of sodium citrate was added under constant stirring. A faint grey color appeared for approximately 1 min, after which the solution was heated on the stirring plate for an additional 20 min. Finally, the solution was allowed to cool to room temperature.

#### 4.2.2 Au@4‐MPy NPs

In 100 mL of Au spheres (concentration of 0.25 mm), 50 µL of 1 mm 4‐MPy ethanol solution was added and the mixture was stirred for 20 min to allow the 4‐MPy to interact with the Au surface. Then, the solution was washed with water (8000 rpm, 20 min) to remove the excess of citrate and 4‐MPy.

#### 4.2.3 Yolk@shell NPs

For their obtention the following precursor solutions were prepared: i) 5 mL solution of 2.5 mm of MnCl_2_.4H_2_O, and ii) 5 mL solution of 2.5 mm K_3_[Fe(CN)_6_] and of 2.5 mm CsCl. A total of 1 mL of solution (i) was added slowly into the colloidal Au@4‐MPy NP solution under stirring and at an addition rate of 100 µL per 20 s. After 10 min, 1 mL of sol (ii) was then added with the same addition rate as sol (i). The final mixture was left to stir for 24 h under ambient conditions. The final solution was centrifuged at 9500 rpm for 20 min thrice (using DI water).

#### 4.2.4 Surface Functionalization of Yolk@shell NPs

1 mg of the previous NPs were redispersed in 2 mL of DI water containing 8 mg of SH‐PEG3500‐COOH. The solution was then stirred moderately overnight, followed by two centrifugation steps (9500 rpm, 10 min) to remove excess PEG.

#### 4.2.5 DOX Loading

DOX loading was achieved by adding 1 mg/1 mL of DOX in H_2_O to a solution of yolk@shell(PEG) with equal concentration (1 mg/1 mL). Since DOX·HCl contains equimolar ratios of DOX and HCl, diluted NaOH was added to the solution to neutralize it. The solution was kept in a thermomixer by Thermo Fischer at 2000 rpm for 24 h, protected from light. After incubation, the solution was centrifuged twice at 13000 rpm to remove unloaded DOX. The supernatant containing the unloaded DOX was collected and analyzed by UV‐Vis to calculate the amount of loaded DOX.

#### 4.2.6 DOX‐Release

The release of DOX was studied by redispersing 1 mg of the compound in 1 mL of PBS at pH 5 or pH 7. An initial aliquot was taken at time zero to establish a baseline for DOX release. At various time intervals, the samples were centrifuged at 13500 rpm for 2 min, and the supernatant was collected for analysis. DOX concentration in the supernatant was measured by UV‐Vis spectroscopy at 480 nm. After each measurement, the precipitate was redispersed in fresh PBS to continue the release study. The cumulative DOX release was plotted as a function of time.

#### 4.2.7 MnFe@Au Hollow Nanoparticles

5 mL aqueous solutions containing 1.5 mg of KAu(CN)_2_ and different concentrations of sodium citrate were prepared. To these solutions, 200 µL of the following precursor solutions were added: i) 2.5 mm MnCl_2_·4H_2_O, and ii) 2.5 mm K_3_[Fe(CN)_6_] with 2.5 mm CsCl. The final mixtures were stirred for 24 h under ambient conditions. The resulting products were collected by centrifugation at 9500 rpm for 20 min, repeated three times, with DI water used for washing.

### 4.3 Physical Characterization

UV‐vis absorption spectra were recorded on Thermofischer multiskan skyhigh microplate spectrophotometer from 300 to 900 nm range, using Thermoscientific clear flat‐bottom immune nonsterile 96‐well plates. 200 µL of the sample was collected in well plates for these measurements. Dynamic Light Scattering (DLS) were performed with a Zetasizer Nano ZS instrument (Malvern Instruments Ltd.). Transmission Electron Microscopy studies carried Hitachi HT7800 120 kV and a Technai G2 F20 operating at 200 kV. Samples were prepared by dropping suspensions on lacey formvar/carbon copper grids (300 mesh). The size distribution was determined by manually counting particles from TEM images using ImageJ software. Attenuated total reflectance Fourier‐transform Infrared Spectroscopy spectra were collected in an Agilent Cary 630 FTIR spectrometer in the 4000–500 cm^−1^ range in the absence of KBr pellets. Powder X‐Ray Diffraction patterns were collected in a PANalytical Empyrean diffractometer using copper radiation (Cu Kα = 1.5418 Å) with a PIXcel detector, operating at 40 mA and 45 kV at room temperature. Thermogravimetric analyses were carried out using a Mettler Toledo TGA/SDTA 851e model operating in the 50°C–550°C range in air. Samples were analyzed by XPS using a KALPHA Thermo Scientific spectrometer. All spectra were collected using Al Kα radiation (1486.6 eV), monochromatized by a twin crystal monochromator, yielding a focused X‐ray spot (elliptical in shape with a major axis length of 400 µm) at 30 mA and 2 kV. The alpha hemispherical analyzer operated in constant energy mode with survey scan pass energies of 50 eV to selectively measure the elements. XPS data were analyzed with Avantage software. A background function was used to approximate experimental backgrounds. Charge compensation was achieved with the system flood gun that provides low‐energy electrons and low‐energy argon ions from a single source.

### 4.4 Cell Culture Experiments

#### 4.4.1 Cytotoxicity Assay

NP toxicity in A549 (ATCC, catalog number: CCL‐185) and Hela (ATCC, catalog number: CCL‐2) was studied using an MTT assay with different samples and controlled concentrations. First, cells were trypsinized, counted, and resuspended in complete medium to achieve 1 × 10^5^ cells/mL concentration. Using a multichannel pipette, 100 µL of this suspension was dispensed into each well of a 96‐well plate. The plates were incubated at 37°C in a humidified atmosphere containing 5% CO_2_ for 24 h to facilitate cell attachment and growth. After, a positive control (DMSO) and various concentrations of the test samples were prepared and placed into a multi‐well reservoir for easy dispensing. Wells were alternated between experimental groups across the plate to minimize positional effects. The culture medium in the 96‐well plates was then carefully aspirated, and 50 µL of the prepared samples were added to the respective wells. The plates were then incubated at 37°C in a 5% CO_2_ atmosphere for 24 h. The MTT stock solution (5 mg/mL) was prepared in PBS, vortexed, sonicated for 5 min, sterilized by filtration through a 0.22 µm filter, and stored at −20°C protected from light. Prior to use, the MTT solution was diluted 1:10 in complete medium. At the designated time point, the medium in the wells was aspirated, and 50 µL of MTT working solution was added. Then, the plates were incubated for 2 h at 37°C in a 5% CO_2_ atmosphere. Following incubation, the MTT solution was aspirated, and 100 µL of isopropanol was added to each well to dissolve the formazan crystals. The plates were gently shaken to ensure complete dissolution. Absorbance was measured at 570 nm using a microplate reader, with a reference wavelength of 650 nm recorded to correct background absorbance.

#### 4.4.2 Cellular Uptake

For both transmission electron microscopy (TEM) and fluorescence microscopy studies, cells were seeded at a density of 40,000 cells/mL onto chamber slides (Nunc Lab‐Tek, Thermo Fisher Scientific) and allowed to adhere for 24 h. Cells were maintained in Dulbecco's Modified Eagle Medium (DMEM; Gibco, Thermo Fisher Scientific, USA) supplemented with 10% fetal bovine serum (FBS; Sigma–Aldrich, USA) and 1% penicillin–streptomycin (Gibco). Afterward, the culture medium was replaced with fresh DMEM containing 50 µg/mL NPs for 4 h. Control samples received medium without NPs.

Following NP exposure, cells designated for TEM analysis were washed twice with PBS (pH 7.4) to remove unbound NPs and culture medium residues. Samples were then fixed with 2.5% glutaraldehyde for 30 min at room temperature, post‐fixed with 2% osmium tetroxide, and embedded in LR‐White resin. Ultrathin sections (60–90 nm) were obtained using a Leica Ultracut UC7 ultramicrotome equipped with a diamond knife. Finally, the sections were contrasted with 2% uranyl acetate and Reynolds’ lead citrate solution.

For fluorescence imaging, cells were washed twice with PBS (pH 7.4) after NP exposure to remove non‐internalized particles. Imaging was performed using a Zeiss LSM 980 confocal laser scanning microscope equipped with a 10× objective and controlled by ZEN software. Fluorescence was recorded using an excitation wavelength of 493 nm and emission at 517 nm. Representative images were collected from multiple random fields for qualitative and semi‐quantitative assessment of intracellular NP localization.

#### 4.4.3 In Vitro Laser Irradiation

An MDL‐H‐808/5 W infrared diode laser with a central wavelength of 808 nm was used. The laser operated in continuous wave mode, with a delivered output power of 2 W/cm^2^. The laser output was coupled into a Thorlabs FT038 optical fiber to ensure precise and uniform light delivery to the NPs.

#### 4.4.4 In Vitro Photothermal Detection

Photothermal heating profiles were collected using the lascar El‐USB‐TC‐LCD thermocouple USB data logger connected to a type K thermocouple.

## Author Contributions

The manuscript was written through contributions of all authors. All authors have given approval to the final version of the manuscript.

## Conflicts of Interest

The authors declare no conflicts of interest.

## Supporting information




**Supporting File**: smll72734‐sup‐0001‐SuppMat.docx.

## Data Availability

The data that support the findings of this study are available from the corresponding author upon reasonable request.
