# Supplementary material for: Gold@MnFe‐Prussian Blue Analog Yolk@Shell Nanoparticles for Light‐Triggered and pH‐Sensitive Drug Release
Source: Small. 2026 Feb 19;22(20):e14869. doi: 10.1002/smll.202514869 (PMC13054203; doi:10.1002/smll.202514869)
Supplement: Supplementary file 1 — Supporting File: smll72734‐sup‐0001‐SuppMat.docx. [file SMLL-22-e14869-s001.docx]

**Gold@MnFe-Prussian Blue Analogue Yolk@Shell Nanoparticles for Light-Triggered and pH-Sensitive Drug Release**

*Shoaib Azeem, Javier Alda-Gómez, Roger Sanchis-Gual,* Marc Coronado-Puchau,* Eugenio Coronado*

S. Azeem, J. Alda, R. Sanchis-Gual, M. Coronado-Puchau, E. Coronado

Instituto de Ciencia Molecular, Universitat de València, Catedrático José Beltrán 2, 46980, Paterna, Spain
E-mail: [roger.sanchis@uv.es](mailto:roger.sanchis@uv.es) ; [marc.coronado@uv.es](mailto:marc.coronado@uv.es)


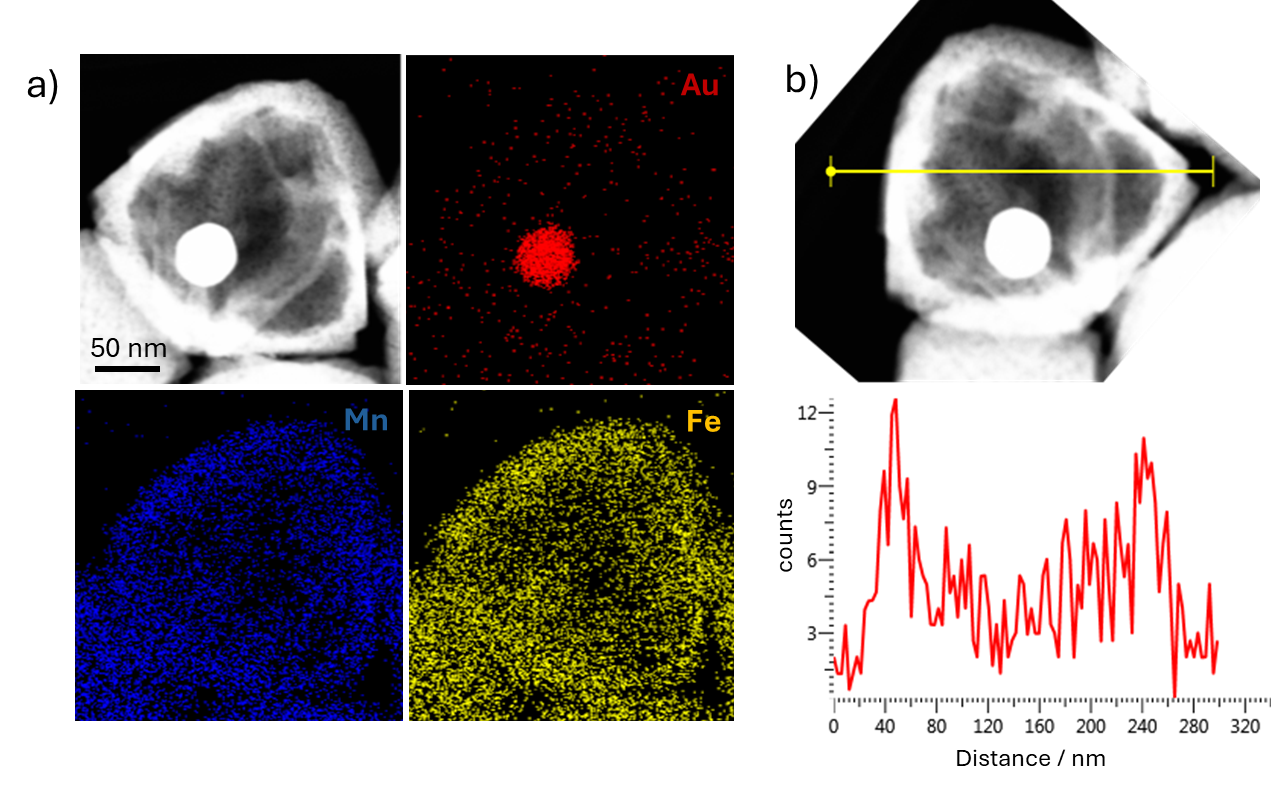


Figure S1. a) EDX mapping of a yolk@shell nanoparticle for the Au, Mn and Fe. b) EDX line profile of Au in the same nanoparticle.


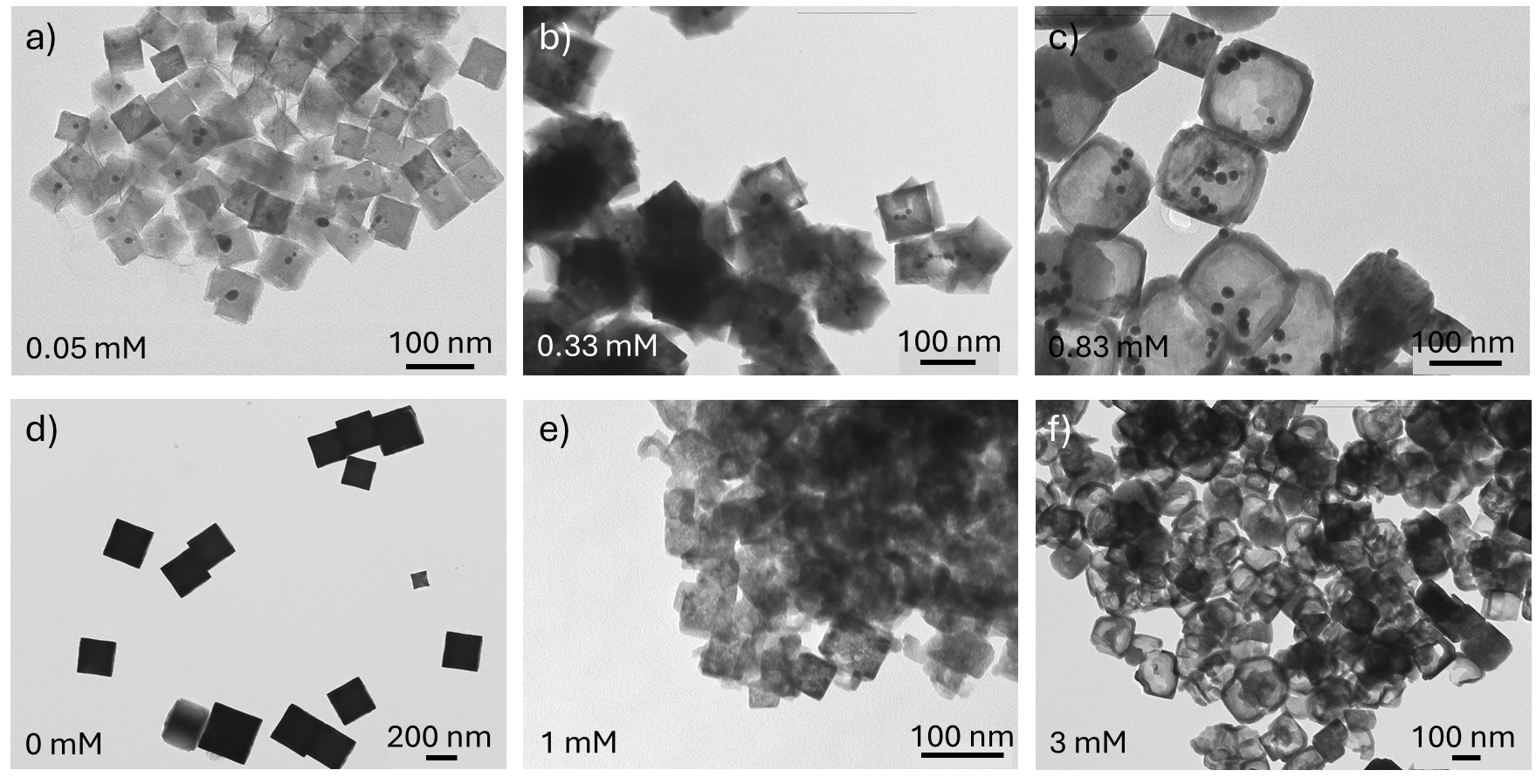


Figure S2. TEM images of (a–c) Au@MnFe-PBA yolk@shells and (d–f) MnFe-PBA nanoparticles (without Au) prepared in solutions with varying sodium citrate concentrations: (a) 0.05 mM, (b) 0.33 mM, (c) 0.83 mM; (d) 0 mM, (e) 1 mM, (f) 3 mM.


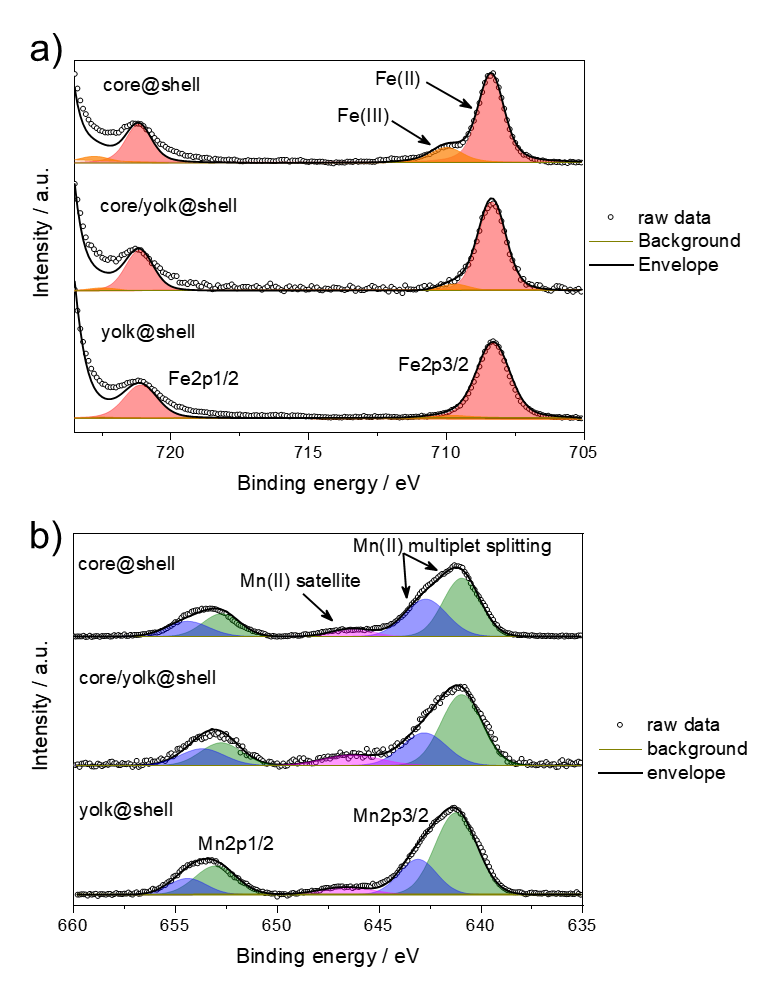


Figure S3. XPS spectra of a) Fe2p and b) Mn2p regions for Au@PBA core@shell (10 min of reaction time), core/yolk@shell (12h of reaction time) and yolk@shell (24h of reaction time) nanoparticles.


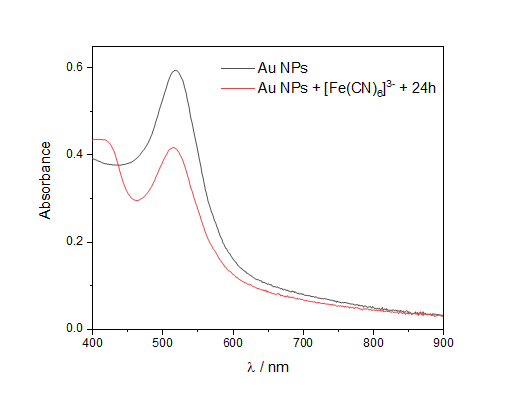


Figure S4. UV-Vis absorbance spectra of AuNPs and of the same nanoparticles after 24 h in 0.25 mM [Fe(CN)_6_]^3-^.


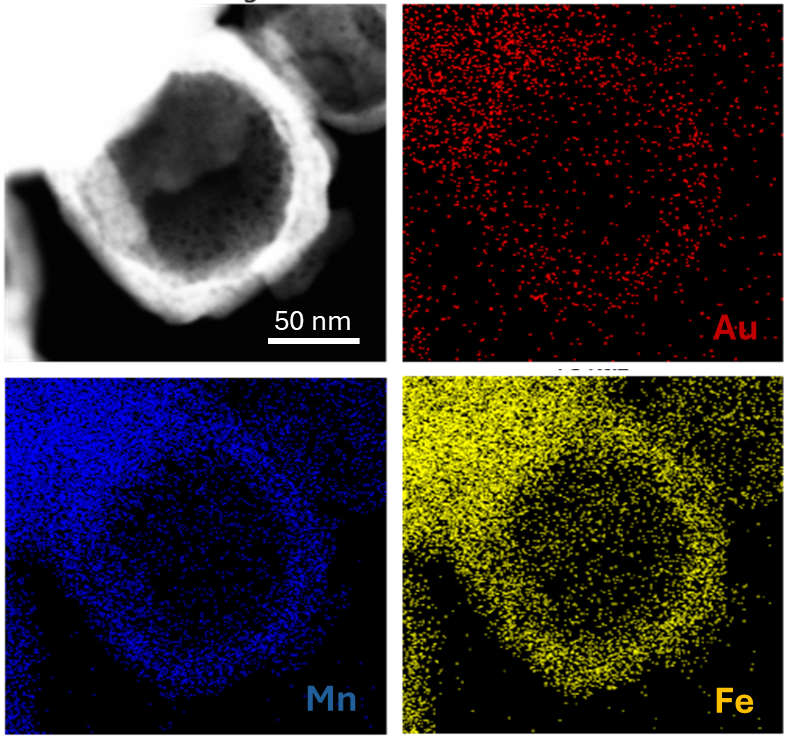


Figure S5. EDX mapping for the Au, Mn and Fe of MnFe-PBA hollow NPs prepared in a solution containing Au(CN)_2_^-^ and sodium citrate.

Figure S6. XPS spectra highlighting the Au4f region of Au NPs with Mn(II) and of yolk@shell nanoparticles.


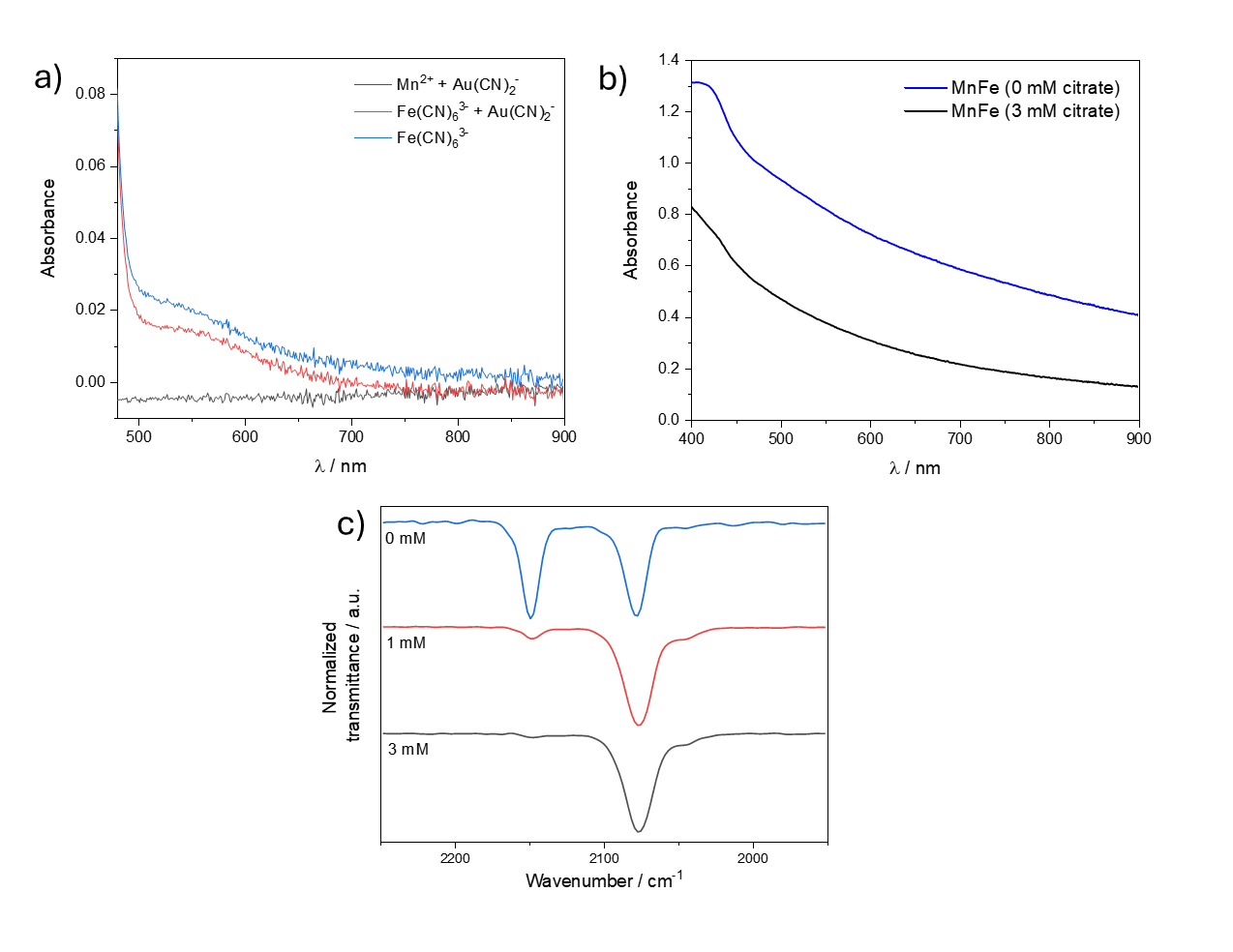


Figure S7. a) UV-Vis absorbance spectra of different solutions containing gold cyanide, manganese and iron cyanide salts. b) UV-Vis absorbance spectra of MnFe(II) and MnFe(III). c) ATR-FTIR spectra highlighting the CN vibration region of MnFe-PBA NPs prepared in a solution with varying sodium citrate concentrations. Peak at 2150 cm^-1^ is attributed to Mn(II)-CN-Fe(III) while peak at 2080 cm^-1^ is attributed to Mn(II)-CN-Fe(II).

Figure S8. PXRD patterns showing the structural comparison between different Au@PBA heterostructures (core@shell and yolk@shell). Peaks marked with an asterisk (*) correspond to metallic Au.


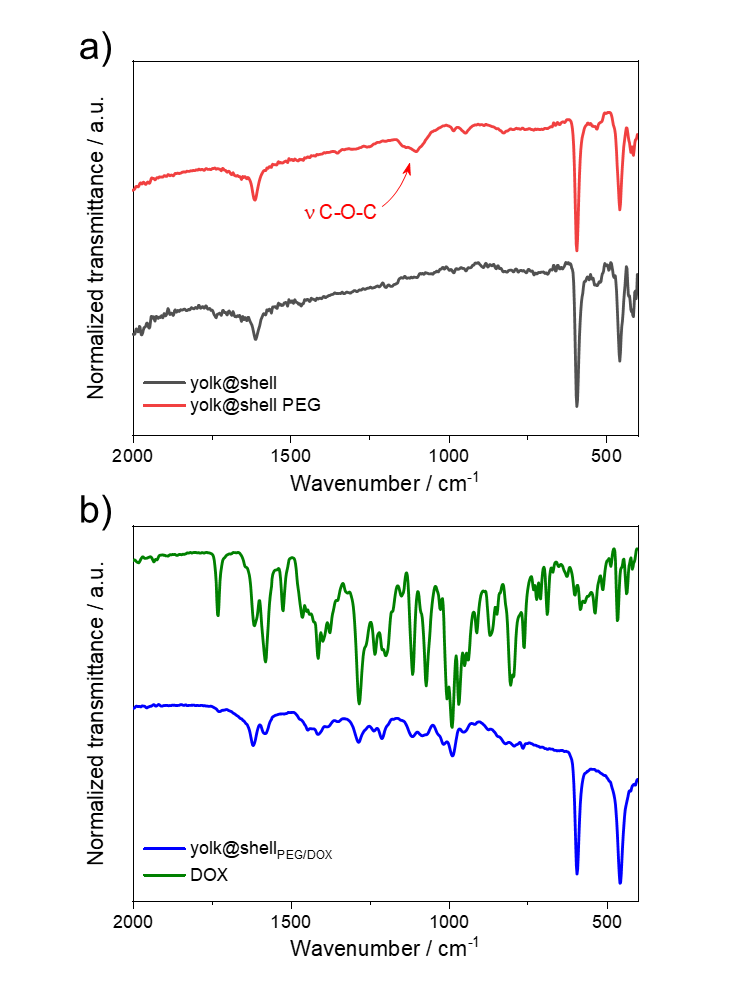


Figure S9. ATR-FTIR spectra of yolk@shell nanoparticles before and after HS-PEG-COOH functionalization and DOX loading.

Figure S10. Thermogravimetric analysis between 50–500 °C comparing yolk@shell nanoparticles, PEG-functionalized yolk@shell, and pure HS-PEG-COOH.

Figure S11. Dynamic Light Scattering measurements of PEG-functionalized yolk@shell nanoparticles over a 48-hour period.

Figure S12. UV–Vis spectra of PEG-functionalized yolk@shell nanoparticles before and after DOX loading.


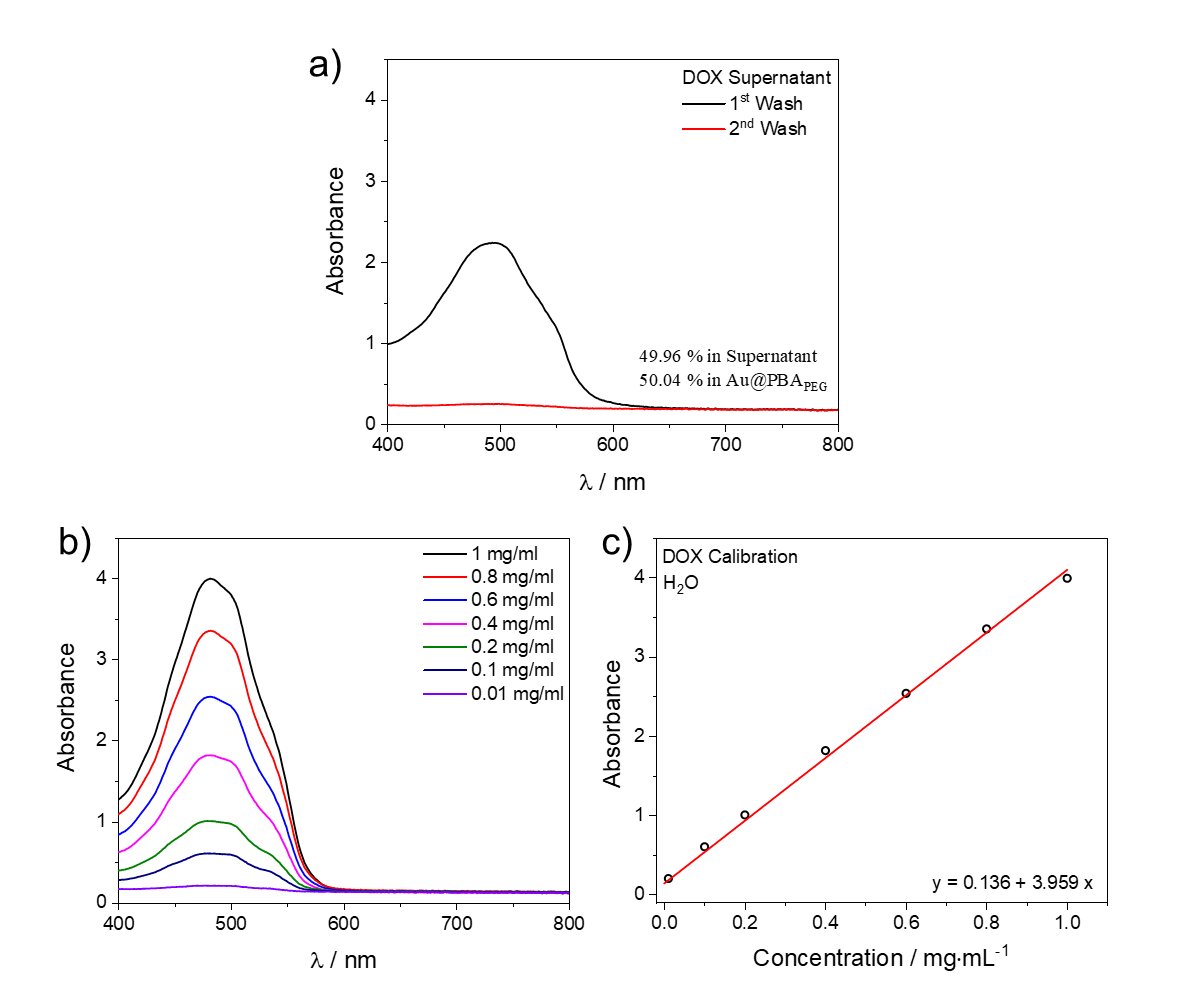


Figure S13. a) UV–Vis absorbance spectra of the supernatant obtained after centrifugation of DOX-loaded yolk@shell nanoparticles post DOX loading in water. (b–c) Calibration curve of DOX concentration versus absorbance in water.


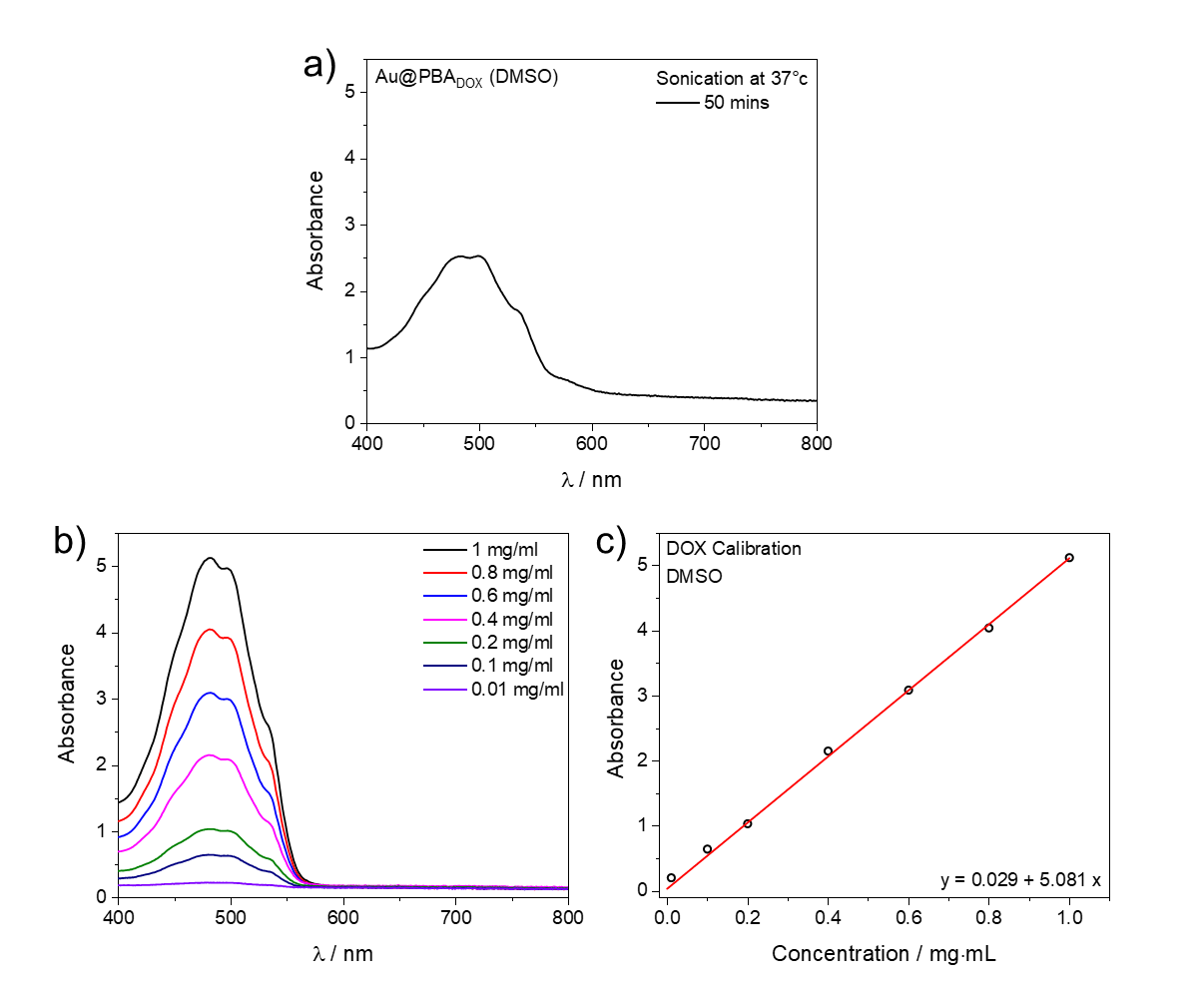


Figure S14. (a) UV–Vis absorbance spectra of the supernatant obtained after complete degradation and centrifugation of DOX-loaded yolk@shell nanoparticles in DMSO. (b–c) Calibration curve of DOX concentration versus absorbance in DMSO.

Figure S15. UV–Vis spectra of the DOX-containing supernatant obtained after centrifugating the core@shell nanoparticles post DOX loading.


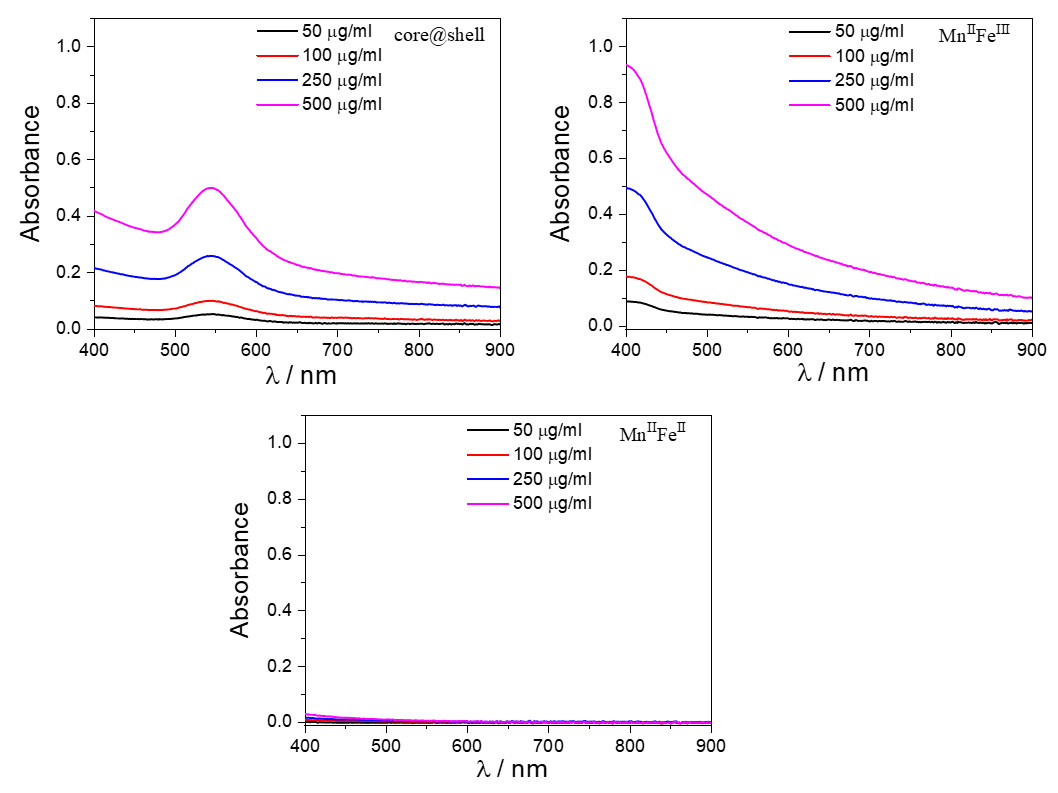


Figure S16. UV-Vis absorbance spectra at varying concentrations for core@shell, Mn(II)Fe(II) and Mn(II)Fe(III) PBA NPs.


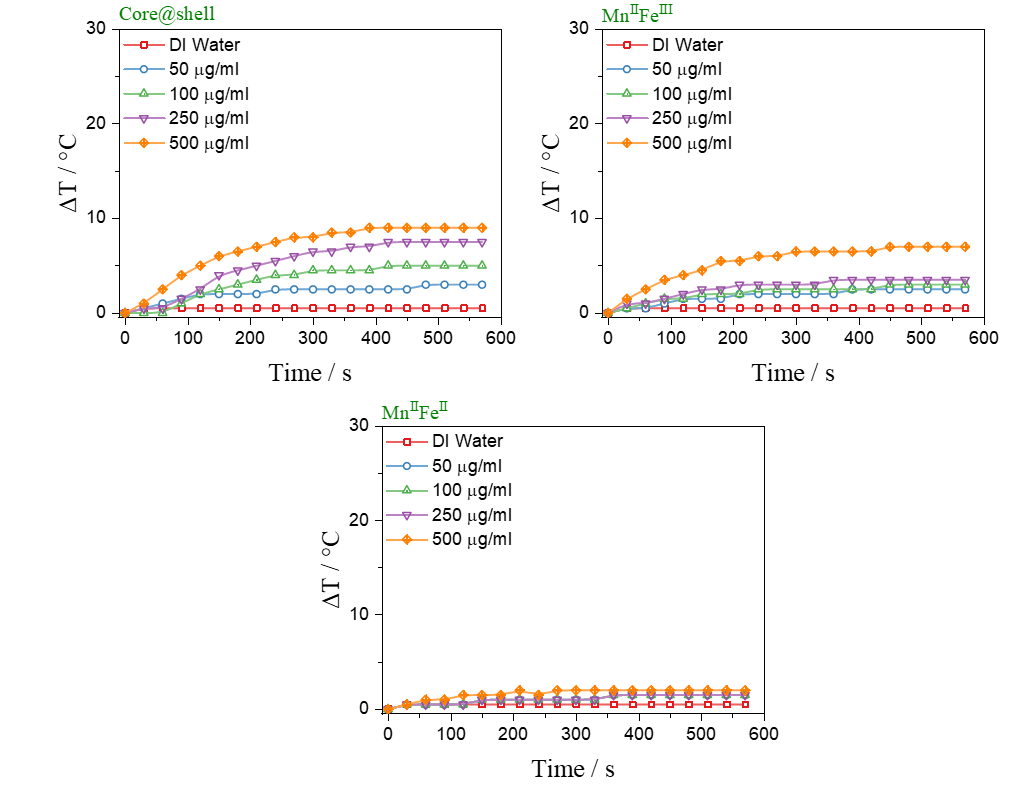


Figure S17. Photothermal heating profiles at varying concentrations under NIR laser irradiation at power density of 2 W/cm^2^ for core@shell, Mn(II)Fe(II) and Mn(II)Fe(III) PBA NPs.

Figure S18. Cumulative DOX release from PEG-functionalized yolk@shell nanoparticles over 24 hours at pH 7 and pH 5, with and without 10 minutes of previous NIR laser irradiation.


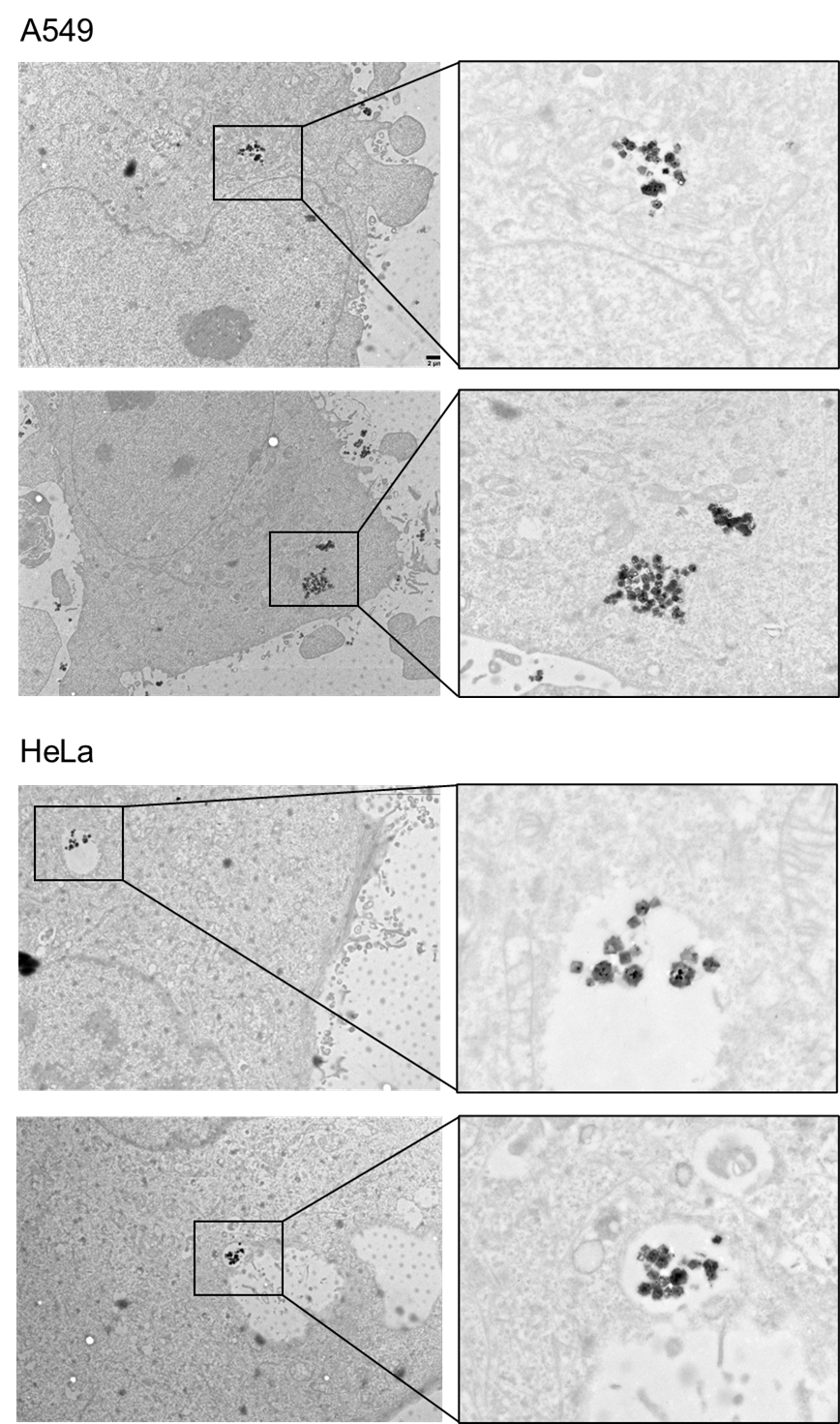


Figure S19. TEM images of PEG-functionalized yolk@shell nanoparticles in A549 and HeLa cells after 4 hours of incubation. Cells were fixed and ultrathin transversal sections were prepared and stained for contrast enhancement.

Figure S20. Cytotoxicity assays: Viability of A549 (left) and HeLa (right) cells after 4 hours of incubation followed by an additional 24 hours, using varying concentrations of PEG-functionalized yolk@shell and DOX-loaded PEG-functionalized yolk@shell nanoparticles. Data are presented as mean + SD of 2 biological replicates, each measured in triplicate.
